# Reviewer acknowledgement 2014

**DOI:** 10.1186/s12947-015-0003-x

**Published:** 2015-02-25

**Authors:** Eugenio Picano, Rosa Sicari

**Affiliations:** CNR Institute of Clinical Physiology, Pisa, Italy

## Abstract

The Editors of *Cardiovascular Ultrasound* would like to thank all of our reviewers who have contributed to the journal in Volume 12 (2014) and whose valuable support is fundamental to its success.

**Eustachio Agricola**

Italy

**Giovanni Donato Aquaro**

Italy

**Dan Berkowitz**

United States of America

**Elisabetta Bianchini**

Italy

**Adrian Borges**

Germany

**Alberto Bouzas-Mosquera**

Spain

**Joan Briller**

United States of America

**Rosa Maria Bruno**

Italy

**Filippo Cademartiri**

Netherlands

**Matteo Cameli**

Italy

**Jelena Celutkiene**

Lithuania

**Annabel Chen-Tournoux**

Canada

**Quirino Ciampi**

Italy

**Rosario Cianci**

Italy

**Maja Cikes**

Croatia

**Giovanni Cioffi**

Italy

**Roberto Copetti**

Italy

**Bernard Cosyns**

Belgium

**Carlos Cotrim**

Portugal

**Giovannide Simone**

Italy

**Victoria Delgado**

Netherlands

**Santo Dellegrottaglie**

Italy

**Sonia Eiras Penas**

Spain

**Carlos Escobar**

Spain

**Francesco Faita**

Italy

**Silvia Favilli**

Italy

**Covadonga Fernández-Golfín**

Spain

**Mark Fridberg**

Canada

**Andrzej Gackowski**

Poland

**Maurizio Galderisi**

Italy

**Lorenzo Galletti**

Italy

**Luna Gargani**

Italy

**Deepak Gupta**

United States of America

**Judy Hung**

United States of America

**Satoru Iwashima**

Japan

**Mark Jermy**

New Zealand

**Jaroslaw Kasprzak**

Poland

**Hyung-Kwan Kim**

Korea, South

**Fabian Knebel**

Germany

**Magdalena Kostkiewicz**

Poland

**Jonathan Lindner**

United States of America

**Piotr Lipiec**

Poland

**Mario Lorenz**

Germany

**Julien Magne**

Belgium

**Marco Magnoni**

Italy

**Lawrence Melniker**

United States of America

**David Messika-Zeitoun**

France

**Jadwiga Moll**

Poland

**Maria Lorenza Muiesan**

Italy

**Sung-Ji Park**

Korea, South

**Gianni Pedrizzetti**

Italy

**Leopoldo Perez de Isla**

Spain

**Pasquale Perrone Filardi**

Italy

**Philippe Pibarot**

Canada

**Alessandro Pingitore**

Italy

**Roberto Pontremoli**

Italy

**Bogdan Alexandru Popescu**

Romania

**Lorenza Pratali**

Italy

**Mario Previtali**

Italy

**Giacomo Pucci**

Italy

**Min Qu**

United States of America

**Daniele Rovai**

Italy

**Taisto Sarkola**

Finland

**André Schmidt**

Brazil

**Roxy Senior**

United Kingdom

**Walter Serra**

Italy

**Jorge Solis**

Spain

**Miguel Sousa Uva**

Portugal

**Francesco Stea**

Italy

**Kjetil Steine**

Norway

**Ariane Testuz**

Switzerland

**Lidia Tomkiewicz-Pajak**

Poland

**Pierre-Jean Touboul**

France

**Albert Varga**

Hungary

**Cecilia Vecoli**

Italy

**Zvi Vered**

Israel

**Susan Wiegers**

United States of America

